# Targeting Mcl-1 Degradation by Bergenin Inhibits Tumorigenesis of Colorectal Cancer Cells

**DOI:** 10.3390/ph16020241

**Published:** 2023-02-06

**Authors:** Yu Gan, Xiaoying Li, Shuangze Han, Li Zhou, Wei Li

**Affiliations:** 1 Department of Radiology, The Third Xiangya Hospital, Central South University, Changsha 410013, China; 2 Department of Pathology, National Clinical Research Center for Geriatric Disorders, The Third Xiangya Hospital, Central South University, Changsha 410008, China; 3 Cell Transplantation and Gene Therapy Institute, The Third Xiangya Hospital, Central South University, Changsha 410013, China

**Keywords:** bergenin, colorectal cancer, Mcl-1, ubiquitination, apoptosis

## Abstract

Myeloid leukemia 1 (Mcl-1) is frequently overexpressed in human malignancies and emerged as a promising drug target. In this study, we verified the inhibitory effect of bergenin on colorectal cancer cells both in vivo and in vitro. In an in vitro setting, bergenin significantly reduced the viability and colony formation and promoted apoptosis of CRC cells dose-dependently. Bergenin decreased the activity of Akt/GSK3β signaling and enhanced the interaction between FBW7 and Mcl-1, which eventually induced Mcl-1 ubiquitination and degradation. Using the HA-Ub K48R mutant, we demonstrated that bergenin promotes Mcl-1 K48-linked polyubiquitination and degradation. In vivo studies showed that bergenin significantly reduced tumor size and weight without toxicity to vital organs in mice. Overall, our results support the role of bergenin in inhibiting CRC cells via inducing Mcl-1 destruction, suggesting that targeting Mcl-1 ubiquitination could be an alternative strategy for antitumor therapy.

## 1. Introduction

Colorectal cancer (CRC) is the most common malignancy of the digestive system, which ranks second in the list of cancer-related causes of death worldwide [1]. The current treatment options for CRC are surgery, chemotherapy, and radiotherapy [2]. However, developing new treatment options is particularly important due to the disadvantages of frequent recurrence, systemic toxicity, unpredictable congenital disorders, and acquired drug resistance [3,4,5].

Aberrant activation of the Bcl-2 gene is an early event in colorectal tumorigenesis, inhibiting apoptosis and promoting tumor progression in vivo [6]. Myeloid leukemia 1 (Mcl-1), an antiapoptotic member of the Bcl-2 family [7], inhibits mitochondrial outer membrane permeabilization by binding to pro-apoptotic Bcl-2 proteins and preventing cytochrome c release and activation of the caspase cascade [8]. Upregulation of Mcl-1 expression is observed in most solid tumors and hematological malignancies [9,10]. Overexpression of the Mcl-1 protein or amplification of the Mcl-1 gene in malignant cells can prevent apoptosis and reduce sensitivity to commonly used anticancer drugs [11], making Mcl-1 a potential drug target for cancer.

Bergenin, a dihydroisocoumarin derivative, is a C-glucoside of 4-O-methyl gallic acid, initially extracted from plants of the genus Bergenia [12] with anti-inflammatory [13], antitumor [14], and anti-hyperuricemic [12] multiple biological activities. It was shown that 11-O-galloyl bergenin can induce the downregulation of antiapoptotic protein expression in human osteosarcoma cells [15]. However, the exact mechanism of its action has not been fully elucidated.

In the present study, the effects of bergenin were investigated on the inhibition of CRC cell proliferation, and the underlying mechanisms were determined. The results suggest that bergenin is a potential antitumor reagent that can be used in CRC therapy.

## 2. Results

### 2.1. Bergenin Inhibits the Proliferation of Human CRC Cells

**Figure 1:**
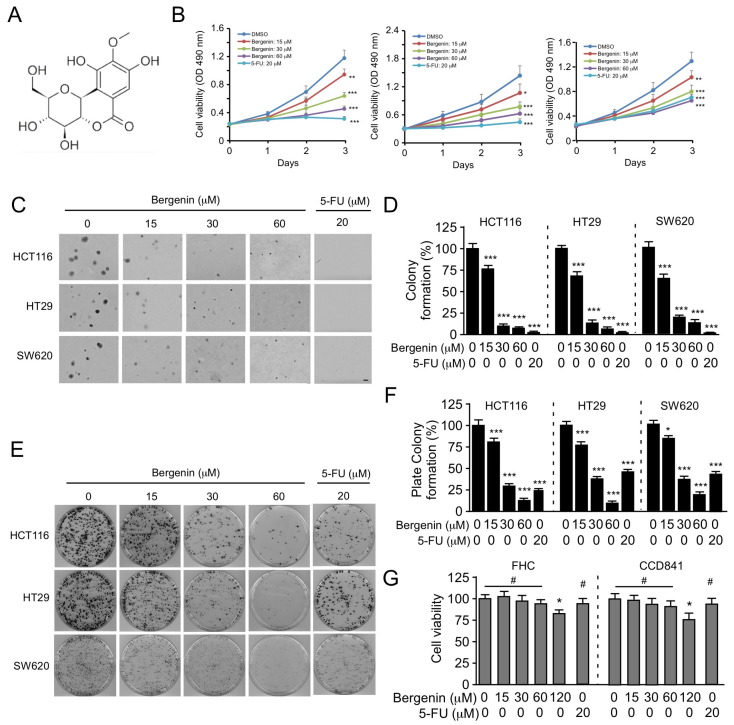
Bergenin inhibits proliferation of CRC cells. The chemical structure of bergenin is shown in (**A**). (**B**) Cell viability analysis of HCT116 (left), HT29 (middle), and SW620 (right) cells was evaluated using the MTS assay following bergenin or 5-Fu treatment. 5-Fu was used as positive control. Data are represented as mean ± SD (n = 3). * *p* < 0.05, ** *p* < 0.01, *** *p* < 0.001. (**C**,**D**) Colony formation of bergenin-treated or 5-Fu-treated HCT116, HT29, and SW620 cells was analyzed by the soft agar method. 5-Fu was used as positive control. (**C**) Colony formation images. Scale bar, 200 μm. (**D**) Quantification. Data are represented as mean ± SD (n = 3). *** *p* < 0.001. (**E**,**F**) The plate colony formation of HCT116, HT29, and SW620 cells following bergenin or 5-Fu treatment. 5-Fu was used as positive control. (**E**) Colony formation images. (**F**) Quantification. Data are represented as mean ± SD (n = 3). * *p* < 0.05, *** *p* < 0.001. (**G**) Cell viability of immortalized FHC and CCD841 non-tumor cells following their treatment with bergenin or 5-Fu for 72 h was analyzed separately by the MTS method. 5-Fu was used as positive control. Data are represented as mean ± SD (n = 3). * *p* < 0.05, #, not statistically significant. CRC, colorectal cancer; MTS, (3-(4,5-dimethylthiazol-2-yl)-5-(3-carboxymethoxyphenyl)-2-(4-sulfophenyl)-2H-tetrazolium; 5-Fu; 5-fluorouracil.

Bergenin (Figure 1A), a natural product, has experimentally exhibited multiple pharmacological activities [16]. To determine the antitumor effects of bergenin on human CRC, the HCT116, HT29, and SW620 cells were treated with this compound. MTS assays indicated that bergenin could significantly inhibit the viability of the CRC cell lines dose-dependently (Figure 1B). The survival of all tested CRC cells decreased by >50% following 72 h of bergenin treatment. Anchorage-independent colony formation and plate colony formation assays indicated that bergenin dose-dependently inhibited the clonogenic (Figure 1C,D) and colony formation ability (Figure 1E,F) of HCT116, HT29, and SW640 cells. Following analysis of the data from the plate colony formation assay, it is evident that the colony formation of human CRC cell lines was reduced by >50% following treatment with 30 μM bergenin. The formation rate could be reduced by >75% following treatment with 60 μM bergenin, which was similar to the effect caused by treatment of the cells with 20 μM 5-fluorouracil. These results suggested that bergenin could inhibit the growth of CRC cells. The effects of bergenin on the immortalized colorectal epithelial cells FHC and CCD841 were assessed, and the data indicated a lack of significant decrease in cell viability, even when the concentration of bergenin reached 60 μM (Figure 1G). These findings suggested that bergenin exhibited an antitumor effect on human CRC cells with no significant toxicity on immortalized non-tumor cells.

### 2.2. Bergenin Promotes Mcl-1 Degradation and Ubiquitination

**Figure 2:**
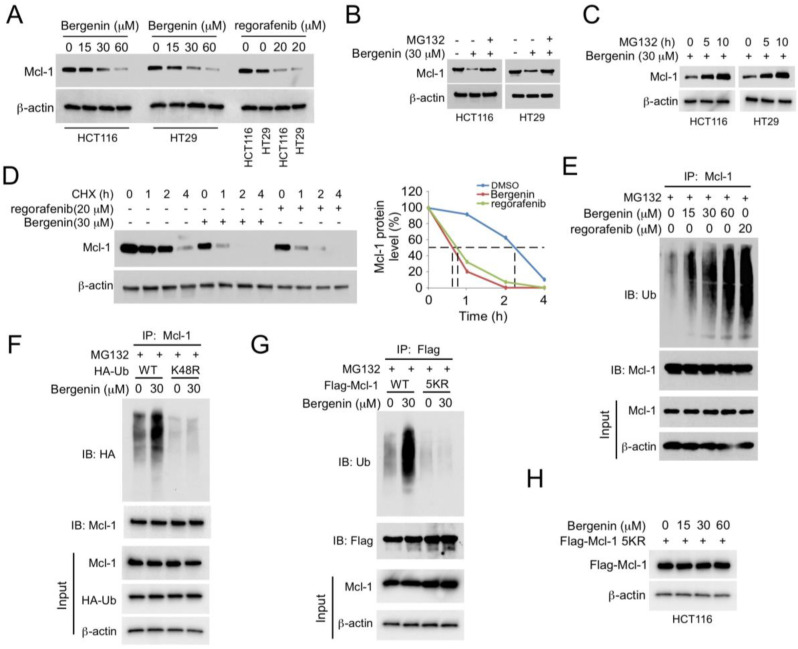
Bergenin induces Mcl-1 degradation. (**A**) Bergenin−treated or regorafenib−treated HCT116 and HT29 were cultured for 48 h. WCE were collected for IB analysis. Regorafenib was used as positive control. (**B**) Bergenin−treated HCT116 and HT29 were cultured for 48 h, followed by culture with 20 µM MG132 for an additional 10 h. WCE were collected for IB analysis. (**C**) Bergenin−treated HCT116 and HT29 cells were cultured for 48 h, followed by culture with 20 µM MG132 for different time points. WCE were collected for IB analysis. (**D**) Bergenin−treated or regorafenib−treated HCT116 and HT29 cells were cultured for 48 h, followed by culture with CHX for different time points. The WCE were collected for IB analysis. Regorafenib was used as positive control. (**E**) Bergenin−treated or regorafenib−treated HCT116 and HT29 cells were cultured for 48 h, followed by culture with 20 µM MG132 for 10 h. WCE were collected, immunoprecipitated with Mcl−1 antibody, and analyzed for Mcl−1 ubiquitination. Regorafenib was used as positive control. (**F**) HCT116 cells were transfected with the HA−Ub wild type or the K48R mutant sequences for 24 h, incubated with bergenin for 48 h, followed by culture with 20 µM MG132 for 10 h. WCE analysis was performed according to the Mcl−1 ubiquitination analysis. (**G**) HCT116 cells were transfected with Flag−Mcl−1 WT or the 5KR mutant sequence for 24 h, incubated with bergenin for 48 h, followed by incubation with 20 µM MG132 for 10 h. Mcl−1 ubiquitination analysis for WCE. (**H**) HCT116 cells were transfected with Flag−Mcl−1 WT or the 5KR mutant for 24 h and incubated with bergenin for 48 h. WCE were collected for IB analysis. WCE, whole cell extracts; IB, immunoblotting; CHX, cycloheximide; Mcl−1, myeloid leukemia 1; WT, wild−type.

The results of the western blotting analysis indicated that bergenin could downregulate the expression levels of Mcl-1 (Figure 2A). This finding was similar to the effect noted by the known tyrosine kinase inhibitor regorafenib which can promote the degradation of Mcl-1 [17]. To further investigate the mechanism of bergenin action, HCT116 and HT29 cells were treated with MG132 for 48 h, and the whole-cell extracts were subsequently extracted for immunoblot analysis. The results indicated that the proteasome inhibitor MG132 could restore Mcl-1 levels in a time-dependent manner (Figure 2B,C). Moreover, the half-life of Mcl-1 was significantly lower following treatment with bergenin than that of the control group (Figure 2D), indicating that the decrease in Mcl-1 levels caused by bergenin was associated with protein degradation. The results of RT-qPCR corroborate this view that the mRNA level of Mcl-1 was unchanged (Supplementary Figure S1A). Ubiquitination analysis indicated that treatment of the cells with 60 μM bergenin could achieve similar degradation of Mcl-1 levels to that noted by 20 μM of regorafenib treatment. In addition, bergenin increased the ubiquitination of Mcl-1 in HCT116 cells in a dose-dependent manner (Figure 2E). We further constructed K-only construct series (K6, 11, 27, 29, 33, 48, 63) to unbiasedly figure out all Ks responsible for Mcl-1 degradation upon bergenin treatment and found that bergenin promoted K48-linked polyubiquitinatoion of Mcl-1 (Supplementary Figure S1B). The endogenous ubiquitination analysis with the K48-linkage specific polyubiquitin rabbit mAb (#12805, CST) further confirmed that Mcl-1 ubiquitination was K48-linked (Supplementary Figure S1C). The exogenous (Figure 2F) co-IP experiments also demonstrated that the ubiquitin K48R mutant reduced bergenin-promoted Mcl-1 ubiquitination. Five lysine residues, including K5, K40, K136, K194, and K197, are dominant sites required for Mcl-1 ubiquitination and degradation [18]. Therefore, the flag-Mcl-1 5KR (all these five lysine residues were mutated to arginines) mutant was used to determine whether these sites were required for the bergenin-induced Mcl-1 degradation. The ubiquitination results indicated that the bergenin-induced ubiquitination of Mcl-1 was significantly reduced in this mutant (Figure 2G). Moreover, although bergenin reduced Mcl-1 protein levels in HCT116 cells, it did not reduce flag-Mcl-1 protein levels in the 5KR mutant (Figure 2H). These results suggested that bergenin promoted Mcl-1 ubiquitination and degradation.

### 2.3. (FBW7) Is Required for Bergenin-Induced Ubiquitination of Mcl-1

**Figure 3:**
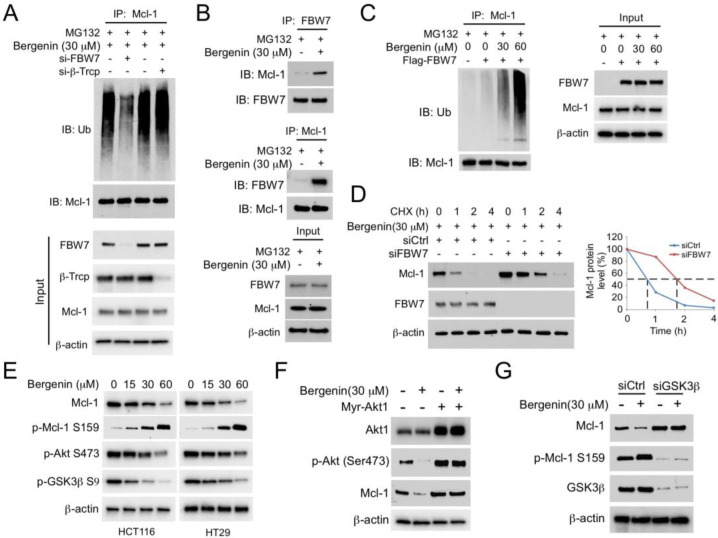
FBW7 is required for bergenin−induced Mcl−1 degradation. (**A**) HCT116 cells were transfected with siFBW7 or siβ−TRCP for 24 h, incubated with bergenin for 48 h, and treated with MG132 for 10 h. Mcl−1 ubiquitination analysis for WCE. (**B**) Bergenin−treated HCT116 cells were cultured for 48 h, followed by culture with 20 µM MG132 for 10 h. The WCE were collected for co−IP analysis. (**C**) HCT116 cells were transfected with a Flag−FBW7 construct for 24 h and treated with bergenin for an additional 48 h. MG132 was added to the cell culture medium and maintained for 10 h. The WCE was subjected to Mcl−1 ubiquitination analysis. (**D**) HCT116 cells were transfected with siFBW7 for 24 h and treated with bergenin for an additional 48 h. CHX was added to the cell culture medium for various time points. The WCE was subjected to IB analysis. (**E**) Bergenin−treated HCT116 and HT29 cells were cultured for 48 h and WCEs were collected for IB analysis. (**F**) HCT116 cells were transfected with a Myr−Akt1 construct for 24 h, followed by incubation with bergenin for 48 h. WCE were collected for IB analysis. (**G**) HCT116 cells were transfected with siGSK3β for 24 h and incubated with bergenin for an additional 48 h. WCE were collected for IB analysis. FBW7, F−box/WD repeat-containing protein 7; si, siRNA; co−IP, coimmunoprecipitation; CHX, cycloheximide; IB, immunoblotting.

At least six E3s (Mule, β-TrCP, FBW7, TRIM17, CDC20, and FBXO4) have been shown to ubiquitinate MCL1 [19]. However, only β-TrCP, FBW7, and TRIM17 are reduced in the non-tumor adjacent tissue (Supplementary Figure S2A). The Kaplan Meier survival curve shows that FBW7, but not β-TrCP or TRIM17, is negatively correlated with the overall survival (OS) (Supplementary Figure S2B), indicating that FBW7 plays a crucial role in regulating MCL-1 degradation in CRC cells. Previous studies revealed that the five ubiquitinated lysine residues of Mcl-1 are catalyzed by E3 ligase FBW7 or beta-transducin repeat containing protein (β-TrCP) [18,20]. FBW7 and β-TrCP expressions were inhibited by small interfering RNAs, and ubiquitination analysis was performed. The results indicated that depletion of FBW7, but not of β-TrCP, resulted in a more substantial decrease in bergenin-induced ubiquitination of Mcl-1 (Figure 3A). Subsequently, the association between Mcl-1 and FBW7 was assessed in CRC cells. The Co-IP results revealed that FBW7 was bound to Mcl-1 in HCT116 cells; this interplay was enhanced with bergenin treatment (Figure 3B). The ubiquitination analysis indicated that bergenin dose-dependently enhanced FBW7-mediated Mcl-1 ubiquitination (Figure 3C). Moreover, a knockdown of FBW7 expression resulted in a substantial prolongation of the half-life of Mcl-1 following bergenin treatment (Figure 3D). These data all suggest a crucial role for FBW7 in bergenin-induced Mcl-1 ubiquitination. A recent study has revealed that GSK3β-induced Mcl-1 phosphorylation is necessary for FBW7-mediated ubiquitination [21] It can be observed that the expression of p-Akt and p-GSK3β showed a bergenin dose-dependent decrease, whereas the protein level expression of p-MCL-1 showed the opposite. GSK3β is a critical downstream element of the PI3K/Akt cell survival pathway whose activity can be inhibited by Akt-mediated phosphorylation at Ser9 [22]. Bergenin suppresses Akt, which activates GSK3β and eventually promotes GSK3β-mediated phosphorylation at Ser159 [21], leading to Mcl-1 destabilization. The data of the present study indicated that bergenin suppressed the activation of Akt/GSK3β signaling and increased Mcl-1 phosphorylation in HCT116 and HT29 cells (Figure 3E), whereas overexpression of constitutively active Akt1 (Myr-Akt) impaired bergenin-induced reduction of Mcl-1 expression (Figure 3F). In addition, knockdown of GSK3β expression restored Mcl-1 protein levels in bergenin-treated HCT116 cells (Figure 3G). Taken together, the data support the notion that bergenin can downregulate Mcl-1 by inducing FBW7 in an Akt-GSK3β signaling-dependent manner.

### 2.4. Bergenin Induces Apoptosis in Human CRC Cells

**Figure 4:**
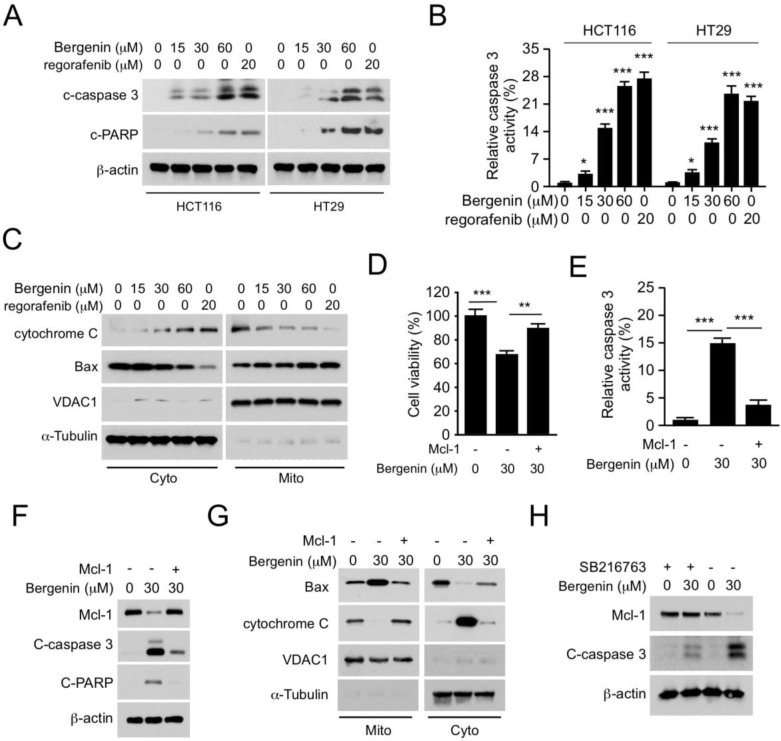
Bergenin induces apoptosis of CRC cells, * *p* < 0.05, ** *p* < 0.01, *** *p* < 0.001. (**A**,**B**) Bergenin−treated or regorafenib−treated HCT116 and HT29 cells were cultured for 48 h. WCE were collected for IB analysis (**A**), and caspase 3 activity was assessed with the caspase−3 assay kit (**B**), Regorafenib was used as positive control. Data are represented as mean ± SD (n = 3). (**C**) HCT116 cells were treated with bergenin or regorafenib for 48 h. Subcellular fractions were isolated and subjected to IB analysis. Regorafenib was used as positive control. (**D**–**G**) HCT116 cells were transfected with Mcl-1 for 24 h, followed by treatment with bergenin for 48 h. The MTS assay was used to assess cell viability (**D**), The caspase−3 assay kit was used to assess caspase 3 activity (**E**), WCE were collected for IB analysis (**F**), and the subcellular fractions was isolated for IB analysis (**G**). Data are represented as mean ± SD (n = 3). (**H**) Bergenin-treated or SB216763−treated HT29 cells were cultured for 48 h. WCE were collected for IB analysis. CRC, colorectal cancer; WCE, whole cell extracts; IB, immunoblotting; MTS, (3−(4,5−dimethylthiazol−2−yl)−5−(3−carboxymethoxyphenyl)−2−(4−sulfophenyl)−2H−tetrazolium.

We further determined whether bergenin affects apoptosis in human CRC cells. Immunoblotting data indicated that bergenin dose-dependently enhanced the expression levels of cleaved-caspase 3 and PARP in HCT116 and HT29 cells (Figure 4A). Caspase 3 activity was also increased in these cells (Figure 4B), indicating that bergenin triggered apoptosis. Furthermore, isolation of subcellular fractions was performed, including cytoplasmic and mitochondrial fractions, to determine if endogenous apoptosis was involved in the mechanism of action of bergenin. Treatment of the cells with the latter increased the release of cytochrome c and reduced its protein levels in the mitochondrial fraction of HCT116 cells. By contrast, the protein expression levels of Bax were decreased in the cytoplasmic fraction and increased in the mitochondrial fraction (Figure 4C). These results indicated that bergenin enhanced the intrinsic apoptosis of human CRC cells at a similar magnitude as that of regorafenib. To determine whether the reduction of Mcl-1 was required for bergenin-induced apoptosis, the Mcl-1 gene was introduced into HCT116 cells, and cell viability and caspase 3 activity were measured by the MTS and caspase-3 assays, respectively. It was observed that the bergenin-induced decrease in cell viability and the increase in caspase 3 activity could be restored by Mcl-1 overexpression (Figure 4D,E). In addition, ectopic overexpression of Mcl-1 could impair the increased expression of cleaved-caspase 3 and PARP in bergenin-treated cells (Figure 4F). The subcellular fraction analysis further confirmed that the increased release of cytochrome c from the mitochondria to the cytoplasm could also be inhibited. These results indicated that a decrease in Mcl-1 expression was necessary to restore the sensitivity of the cells to bergenin (Figure 4G). The GSK-3β inhibitor SB216763 compromised bergenin-induced apoptosis in CRC cells (Figure 4H), suggesting that GSK-3β is critical for bergenin-induced apoptosis in CRC cells.

### 2.5. Mcl-1 Affects the Tumorigenic Properties of Human CRC Cells

**Figure 5:**
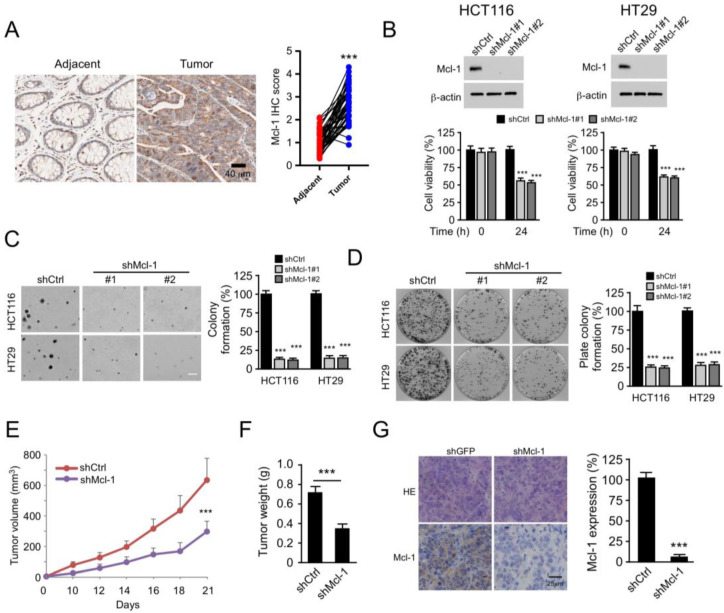
Knockdown of Mcl-1 expression suppresses tumorigenesis of CRC cells, *** *p* < 0.001. (**A**) IHC analysis of Mcl-1 staining in 43 CRC tissues and adjacent non-tumor tissues. Representative images of Mcl-1 IHC staining are shown on the left. The identification results are shown on the right. Scale bar, 40 μm. (**B**) Cell viability of HCT116 (left) and HT29 (right) cells expressing shCtrl or shMcl-1. Top, IB analysis of Mcl-1 expression. Bottom, cell viability as determined by MTS analysis. Data are represented as mean ± SD (n = 3). (**C**) The soft agar assay analysis of the colony formation assay corresponding to HCT116 and HT29 cells transfected with shCtrl or shMcl-1. Data are represented as mean ± SD (n = 3). Scale bar, 200 μm. (**D**) Colony formation of HCT116 and HT29 cells in plates transfected with shCtrl or shMcl-1. Data are represented as mean ± SD (n = 3). (**E**–**G**) Tumor development of shCtrl- or shMcl-1-transfected HCT116 cells in vivo. Measurement of tumor volume (**E**), weight (**F**), and Mcl-1 IHC staining (**G**). E-F, n = 5 (5 mice), (**G**) data are represented as mean ± SD (n = 6); 2 tissue slides per tumor. Scale bar, 25 μm. IHC, immunohistochemical; Mcl-1, myeloid leukemia 1, CRC, colorectal cancer; sh, short hairpin RNA; MTS, (3-(4,5-dimethylthiazol-2-yl)-5-(3-carboxymethoxyphenyl)-2-(4-sulfophenyl)-2H-tetrazolium; IB, immunoblotting.

Evasion of cell death is one of the main hallmarks of cancer [23]. As a key factor in resistance to apoptosis, we next investigated whether Mcl-1 is necessary for the tumorigenic properties of CRC. To determine the differential expression of Mcl-1 in CRC, the expression levels of this protein were compared in 43 CRC samples and adjacent non-cancerous tissues by immunohistochemical staining. The results indicated that the expression levels of Mcl-1 were significantly upregulated in CRC tissues compared with those noted in paired adjacent tissues (Figure 5A). Mcl-1 depleted HCT116 and HT29 stable cell lines were established to determine the role of Mcl-1 in CRC cells. The MTS assay indicated that depletion of Mcl-1 reduced cell viability (Figure 5B). The ability of the cells to divide independently of contact with the extracellular matrix or the neighboring cells is one of the typical characteristics of tumor cells. Therefore, Mcl-1 knockdown HCT116 and HT29 cells were incubated as single cells in soft agar, and the results demonstrated impaired anchorage-independent colony formation potential of CRC lineage cells following knockdown of Mcl-1 expression (Figure 5C). The plate colony formation assay further indicated that the knockdown of Mcl-1 expression reduced the colony formation ability of HCT116 and HT29 cells (Figure 5D). Subsequently, xenograft mouse models, were constructed using HCT116-short hairpin RNA (sh)Ctrl and HCT116-shMcl-1 stable cells. The data indicated that downregulation of Mcl-1 expression inhibited in vivo tumor growth (Figure 5E–G). These results suggested that Mcl-1 was required for CRC growth.

### 2.6. Bergenin Inhibits Tumor Growth In Vivo

**Figure 6:**
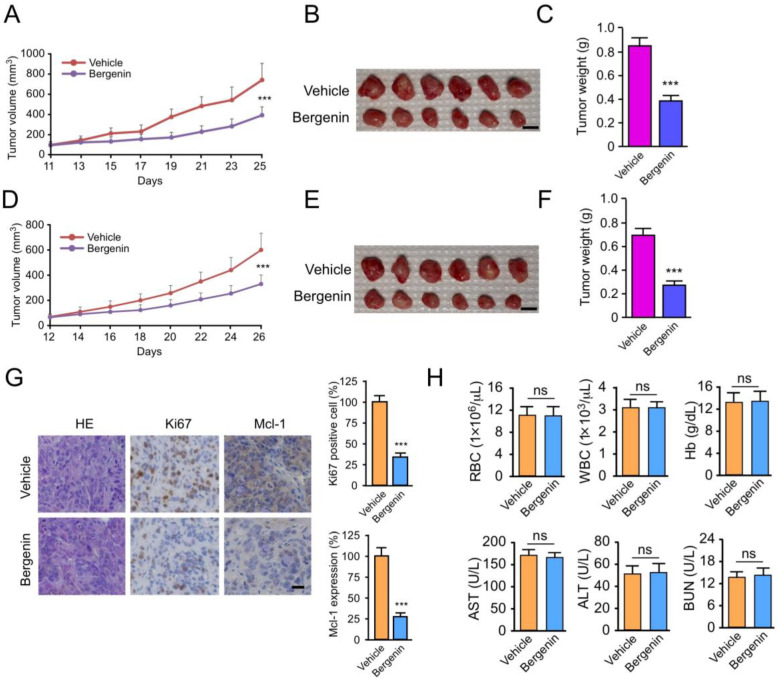
Bergenin inhibits tumor growth in vivo, *** *p* < 0.001. (**A**–**C**) HCT116-derived xenograft tumors were treated with vehicle control or bergenin for different time periods, and subsequently tumor volume (**A**), mass image (**B**), and weight (**C**) were measured separately. Scale bar, 1 cm. Data are represented as mean ± SD (n = 3). (**D**–**F**) Treatment of HT29-derived xenograft tumors with vehicle control or bergenin, followed by measurement of tumor volume (**D**), mass image (**E**), and weight (**F**). Scale bar, 1 cm. Data are represented as mean ± SD (n = 3). (**G**) HCT116-derived xenograft tumors were treated with vehicle control or bergenin, followed by IHC staining and assays for Ki67 and Mcl-1 expression. Scale bar, 25 μm. Data are represented as mean ± SD (n = 6); 2 tissue slides per tumor. (**H**) Blood specimens were analyzed for white blood cells (WBC), red blood cells (RBC), hemoglobin (Hb), alanine aminotransferase (ALT), aspartate aminotransferase (AST), and blood urea nitrogen (BUN) with/without bergenin treatment. Data are represented as mean ± SD (n = 3). IHC, immunohistochemical, Mcl-1, Mcl-1, myeloid leukemia. ns: no significance.

To explore the antitumor activity of bergenin in vivo, HCT116 and HT29 cells were used to establish mouse xenograft models. The data suggested that the administration of bergenin significantly delayed the tumorigenesis of CRC cells. In HCT116-derived xenograft tumors, the average tumor volume of the bergenin-treated group was 383 ± 82 mm^3^, whereas the volume of the vehicle control group was 743 ± 165 mm^3^ (Figure 6A). The tumor weight was reduced by >50% following bergenin treatment (Figure 6B,C). A similar inhibitory effect was also observed in HT29-derived xenograft tumors, and it was shown that bergenin inhibited tumor volume and decreased tumor weight (Figure 6D–F). In addition, IHC staining was used to examine the protein levels of Ki67 and Mcl-1 in the HCT116 xenograft tumors. Data analysis revealed that bergenin downregulated Mcl-1 expression in tumor tissues and significantly decreased the population of Ki67-positive cells (Figure 6G). The blood sample test showed no significant difference in the RBC, WBC, Hb, ALT, AST, and BUN between the vehicle control and the bergenin-treated groups (Figure 6H). Notably, histopathology in mice throughout the study period did not demonstrate significant evidence of bergenin toxicity on vital organ functions, including those of the heart, liver, spleen, kidney, and lung (Supplementary Figure S3), suggesting that bergenin is well-tolerated in vivo.

## 3. Discussion

Bergenin is an isocoumarin derivative with five hydroxyl groups, which has received increased attention due to its multiple and important pharmacological activities. It was shown that bergenin exerts direct antipulmonary fibrotic action by activating the p62-Nrf2 positive feedback loop [24]. It also activates the PI3K/Akt signaling pathway in vivo and in vitro to reduce Parkinson’s disease symptoms [25]. Recently, it was shown that bergenin is a novel galectin-3 inhibitor with potential antitumor activity [26] and inhibits HepG2 cells and decreases the expression of the Bcl-2 family of proteins [27]. In addition, bergenin increased G1 phase arrest, and reduced expression of Ki67, cycling D1, and cycling B1 in bladder cancer cells [28]. 11-O-galloyl bergenin induces autophagy and apoptosis in human osteosarcoma [15]. Piperazine tethered bergenin heterocyclic hybrids disrupted and mitigated the cell cycle progression at the G0/G1 phase in the tongue and oral cancer cell lines [29]. However, the exact mechanism by which bergenin induces apoptosis in tumor cells is still not fully elucidated. The present study reported that bergenin induced apoptosis in human CRC cells partially via Mcl-1 degradation. Based on the in vivo result, bergenin (30 mg/kg) had no significant toxicity to vital organ functions, as reflected by the results of liver, kidney, and bone marrow function tests. The levels (NOAEL) of bergenin with no observed adverse effect from our animal studies were used to estimate the human equivalent dose (HED) using the factor method [30], and the initial dose used for possible translation in humans (60 kg) is 12.78 mg. Of note, the chemical dose of bergenin from preclinical toxicological studies to HED needs further clarification.

Mcl-1 plays a key role in the survival and development of cancer cells [31,32]. As a member of the Bcl-2 family, overexpression of Mcl-1 in cancer cells disrupts the balance between antiapoptotic and pro-apoptotic proteins, leading to a malignant proliferation of tumors [33]. Various cytokines and signaling pathways are involved in the regulation of Mcl-1. For example, the combination of Bcl-2 and PI3Kδ inhibitors can downregulate Mcl-1 expression mediated by Akt/4E-BP-1 inactivation [34]. Overexpression of human mab21L1 gene cDNA in αTN4-1 cells upregulates expression of the pro-survival regulator Mcl−1 during okadaic acid treatment [35]. In addition, miR-148a can directly target ROCK1/c-Met to inhibit Mcl-1 protein expression, thereby reducing angiogenesis and increasing apoptosis in colon cancer cells [36]. Targeting ubiquitination of Mcl-1 is allowed for rapidly eliminating proteins and triggering cell death while responding to various cellular events [37]. Environmental cadmium exposure leads to placental apoptosis and fetal growth restriction through Parkin-induced degradation of Mcl-1 ubiquitination during gestation [38]. Inhibition of BMI-1 also leads to DUB3-dependent Mcl-1 degradation, resulting in enhanced apoptosis of cancer cells [39]. The assessment of the mechanism of action of bergenin revealed that its treatment to CRC cells reduced Akt/GSK3β signaling activity, suggesting that it could promote FBW7-mediated Mcl-1 degradation and ultimately apoptosis in CRC cells. As the regulation of Mcl-1 stability depends on the cell type and stimulus, it is crucial to identify the E3 ligase enzymes that can regulate Mcl-1.

Restoring apoptosis to cancer cells is an important strategy for treating cancer and overcoming resistance to various cancer therapies [40,41]. The importance of MCL-1 in combating resistance to chemotherapy, radiotherapy, and targeted therapies has been well documented [42]. Recent studies showed that mTOR-mediated resistance to PI3Kβ and Akt inhibitors in breast cancer cells can be reversed by inhibiting the protein levels of Mcl-1 [43]. Paclitaxel resistance was also shown to be associated with increased phosphorylation of Bcl-2 and decreased levels of Mcl-1. Inhibition of glutamine uptake in ovarian cancer can resensitize tumor cells to paclitaxel resistance by downregulating the mTORC1/S6K signaling pathway [44]. Our previous study also showed that Skp2 stabilizes Mcl-1 and thus confers radioresistance to colorectal cancer [45], suggesting that targeting Mcl-1 could be an extremely promising strategy for cancer treatment. S63845 was the first MCL-1 inhibitor reported to be effective in tumor models, binding human Mcl-1 at a Kd of 0.19 nM, and was shown to have broad therapeutic applicability in multiple myeloma, lymphoma, leukemia, and primary AML cell groups [46]. NA1-115-7 is the latest type of selective Mcl-1 inhibitor that specifically induces apoptosis in Mcl-1-dependent tumor cells, and two hours of treatment is sufficient to trigger cell death [9]. Natural products with high bioactivity and low toxicity have been widely used in tumor therapy research [47]. Both Allyl isothiocyanate [48] and Pyrethrum extract [49] weere found to have a role in regulating Mcl-1 levels, but the exact mechanism remains to be elucidated. In the present study, the antitumor effects of bergenin were investigated on CRC cells and its mechanism of action was assessed in vitro and in vivo. The results indicated that bergenin significantly inhibited the viability of CRC cells and induced apoptosis in an Mcl-1-dependent manner. The present study suggests that bergenin enhances the interaction of the E3 ubiquitin ligase FBW7 with Mcl-1, eventually promoting Mcl-1 degradation.

Overall, the results provided in this study indicated that bergenin induced apoptosis in CRC cells via FBW7-mediated Mcl-1 ubiquitination. The data provide a new selection strategy for developing novel Mcl-1 inhibitors in anticancer therapy.

## 4. Materials and Methods

### 4.1. Antibodies and Reagents

The natural product bergenin, MG132, 5-Fu, regorafenib, and cycloheximide (CHX) were obtained from Selleck Chemicals (Houston, TX, USA). The transfection reagent Lipofectamine^TM^ 2000 was purchased from Thermo Fisher Scientific Inc. (Waltham, MA, USA). Antibodies against cytochrome c (#11940, 1:1000), Bax (#14796, 1:1000), phosphorylated (p)-GSK3β-Ser9 (#12456, 1:1000), p-Mcl-1-Ser159 (#39224, 1:1000), p-Akt-Ser473 (#4060, 1:1000), Akt (#4691, 1:1000), cleaved-caspase 3 (#9664, 1:1000), β-actin (#3700, 1:10,000), and cleaved-poly (ADP-ribose) polymerase (PARP) (#5625, 1:1000) were obtained from Cell Signaling Technology, Inc. (Beverly, MA, USA). The anti-Ki67 (#ab15580, 1:300) was purchased from Abcam (Cambridge, UK).

### 4.2. Cell Lines and Cell Culture

Human colorectal cancer cell lines, HCT116, HT29, and SW620, and the non-tumor FHC and CCD841 cells, were purchased from the American type culture collection (ATCC, Manassas, VA, USA). Cells were cytogenetically tested and authenticated before being frozen. Each vial of frozen cells was thawed and maintained for 2 months (10 passages). All cells were maintained at 37 °C in a humidified incubator with 5%  CO_2_ according to the ATCC protocols. The cells were cytogenetically tested and authenticated before being frozen. When it is time for passaging, remove the medium from the humidified incubator, rinse with PBS, expose the cells to trypsin, wait 2–3 min, stop the trypsin activity by adding medium containing serum, dissociate the cells, dilute, and re-enter the incubator.

### 4.3. Clinical Tissue Sample Collections

This study was approved (2020-S518) by the Research Ethics Committee of The Third Xiangya Hospital, Central South University. A total of 43 cases of CRC tissues and matched adjacent non-tumor tissues were collected from 43 patients from the Department of Pathology of the Third Xiangya Hospital of Central South University, Changsha, Hunan, China. Patients provided written informed consent. All the patients received no treatment before surgery.

### 4.4. Immunohistochemical (IHC) Staining

The present study was approved by the research ethics committee of the institute of The Third Xiangya Hospital, Central South University. Human CRC tissues and paired paracancerous tissue samples were obtained from the Department of Pathology, The Third Xiangya Hospital. Written informed consent was obtained from all subjects for their participation in the study (n = 43). All patients did not receive any preoperative treatment. The tissue sections of the xenograft tumor tissues were baked at 60 °C for 2 h, dewaxed, and subsequently rehydrated. The sections were immersed in 10 mM (pH 6.0) boiling sodium citrate buffer for 10 min for antigen repair and subsequently treated with 3% H_2_O_2_ for 10 min. The slides were blocked with 10% goat serum albumin for 1 h at room temperature in a humidified chamber, followed by incubation with primary antibodies overnight at 4 °C. The 3,3′-diaminobenzidine substrate was used to visualize the target proteins following 45 min of hybridization with the secondary antibody at room temperature. Hematoxylin was used for counterstaining. The slides were imaged by a light microscope and analyzed using the Image-Pro Plus software (version 6.2).

### 4.5. Protein Preparation and Western Blotting

Whole-cell lysis products were extracted with RIPA buffer containing 10 mM Tris-Cl (pH 8.0), 1 mM EDTA, 0.5 mM EGTA, 1% Triton X-100, 0.1% sodium deoxycholate, 0.1% sodium dodecyl sulfate, and 140 mM NaCl. The lysis products were sonicated and centrifuged at 12,000× *g* for 15 min. The protein concentrations were determined using the BCA analytical reagent (#23228, Thermo Fisher Science, Waltham, MA, USA). The protein samples (20 μg) were separated by 10% SDS-PAGE, transferred to PVDF membranes, and incubated with 5% skimmed milk. After incubation with a primary antibody overnight at 4 °C, the membrane was washed three times with TBS-Tween^®^20 and then incubated with a secondary antibody at room temperature for 1 h. The immunoblot bands were observed using ECL reagents (#34579, Thermo Fisher Scientific, Inc. Waltham, MA, USA).

### 4.6. 3-(4,5-Dimethylthiazol-2-yl)-5-(3-carboxymethoxyphenyl)-2-(4-sulfophenyl)-2H-tetrazolium (MTS) Assay

CRC cells were plated at a density of 2 × 10^3^ cells per well in 100 μL of medium containing 10% FBS in 96-well plates and incubated at 37 °C in a 5% CO_2_ incubator. The cells were treated with various concentrations of bergenin, and cell viability was assessed using the MTS assay (Promega, Madison, WI, USA) according to the instructions provided by the manufacturer.

### 4.7. Ubiquitination Assay

The cells were harvested and lysed with modified RIPA buffer (20 mM NAP, pH 7.4, 150 mM NaCl, 1% Triton, 0.5% sodium deoxycholate, and 1% SDS) containing protease inhibitors and 10 mM N-ethylmaleimide. The lysates were sonicated for 30 s, boiled at 95 °C for 5 min, diluted with RIPA buffer containing 0.1% SDS, and centrifuged at 4 °C (16,000× *g* for 15 min). The supernatant was transferred to a new tube and incubated overnight at 4 °C with Mcl-1 antibody containing protein A-Sepharose beads. Following extensive washing, using 2 × SDS loading buffer to elute the bound proteins, the samples were separated using SDS-PAGE and analyzed by protein blotting.

### 4.8. In Vivo Tumor Growth Assay

All mice were maintained and manipulated according to strict guidelines established by the Institutional Animal Care and Use Committees of Central South University (No. 202009655), Changsha, China. Briefly, 6-week-old thymus-free female BALB/c-Nude mice (Strain NO.D000521) with a body weight of around 16 ± 2 g were purchased from Gempharmatech Co. Ltd., Nanjing, China. Xenograft mice models were generated by suspending colorectal cells HCT116 or HT29 cells (1 × 10^6^) in 100 μL of RPMI-1640 medium and injected into the right flank of nude mice (n = 6). The size of the subcutaneous tumors was calculated and recorded every 2 days using vernier calipers as follows: tumor volume (mm^3^) = (length × width × width/2), and the measurements were repeated three times. Bergenin treatment was initiated when the volume reached around 100 mm^3^ [50]. The drug bergenin for intraperitoneal injection is dissolved in 70% corn oil + 30% PEG400, and the same amount of vehicle control contains 70% corn oil+ 30% PEG400. The tumor-bearing mouse was euthanized by CO_2_ when the average tumor volume of the vehicle group reached 800 mm^3^ (day 25 in the HCT116 and day 26 in the HT29). The fill rate of carbon dioxide is 30% of the chamber volume per minute (3 L/min), and the duration time is 5 min. Death was further confirmed by cervical dislocation. At that time, the tumors were removed and subjected to IHC staining.

### 4.9. Statistical Analysis

Statistical analyses were performed using SPSS (version16.0 for Windows, SPSS Inc, Chicago, IL, USA) and GraphPad Prism 5 (GraphPad 5.0, San Diego, CA, USA). All quantitative data are expressed as mean ± SD of three independent experiments. The Student’s *t*-test or ANOVA was used to assess the differences between the means. *p* < 0.05 was considered to indicate a statistically significant difference.

## Data Availability

Data is contained within the article and Supplementary Materials.

## Supplementary Materials

The following supporting information can be downloaded at: https://www.mdpi.com/article/10.3390/ph16020241/s1, Figure S1: Supplementary figure for result 2.2; Figure S2: Supplementary figure for result 2.3; Figure S3: Supplementary figure for result 2.6; Figure S4: Full gel for Figure.

## Abbreviations

CRC, colorectal cancer; Mcl-1, myeloid leukemia 1; IB, immunoblotting; co-IP, co-immunoprecipitation; IHC, immunohistochemistry; MOMP, mitochondrial outer membrane permeabilization.
