# Supplementary material for: Targeting Mcl-1 Degradation by Bergenin Inhibits Tumorigenesis of Colorectal Cancer Cells
Source: Pharmaceuticals (Basel). 2023 Feb 6;16(2):241. doi: 10.3390/ph16020241 (PMC9965350; doi:10.3390/ph16020241)

Supplementary Figure S1 A, HCT116 and HT29 cells were treated with bergenin for 48 h, the mRNA level of Mcl-1 was examined by qRT-PCR. B, HCT116 cells were co-transfected with Flag-Mcl-1 and His-ubiquitin K-only mutant series (K6, K11, K27, K29, K33, K48, K63, WT) for 24 h, incubated with bergenin for 48 h, followed by culture with 20 μM MG132 for 6 h. Ni-NTA pull-down assay was performed to detect Mcl-1 ubiquitination. C, HCT116 cells were incubated with bergenin for 48 h, followed by culture with 20 μM MG132 for 6 h. Mcl-1 ubiquitination was analyzed with the Ub-K48 specific antibody.

Supplementary Figure S2. A. The expression levels of various E3-ligases such as

β-TCRP、FBW7、TRIM17、FBXO4、CDC20 and Mule mRNA in tumor tissues (COAD and READ) (red box) and non-tumor tissues (grey box) were analyzed by GEPIA (http://gepia.cancer-pku.cn/). *P<0.05. B. Association between FBW7 (left)、β-TCRP (middle) or TRIM17 (right) protein level and overall survival (OS) of CRC patients assessed by Kaplan–Meier survival curves from Proteinatlas database (https:// www.proteinatlas.org/).

Supplementary Figure S3. HE staining of the heart, liver, spleen, lung, and kidney from the vehicle or Bergenin treated mice. Scale bar, 25μm.

Supplementary Figure S1

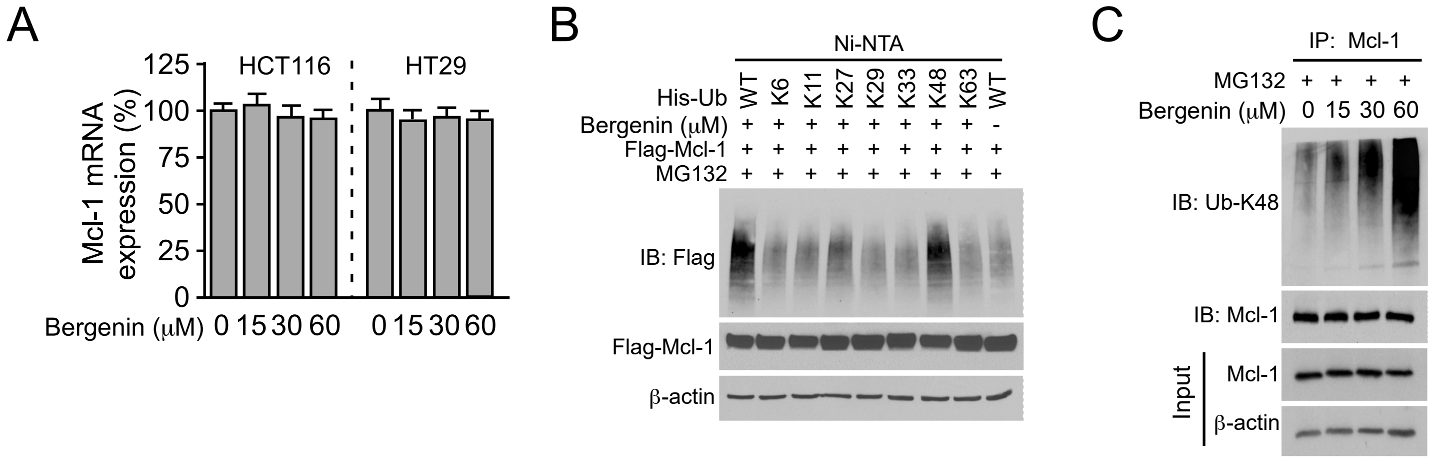

Supplementary Figure S2

A

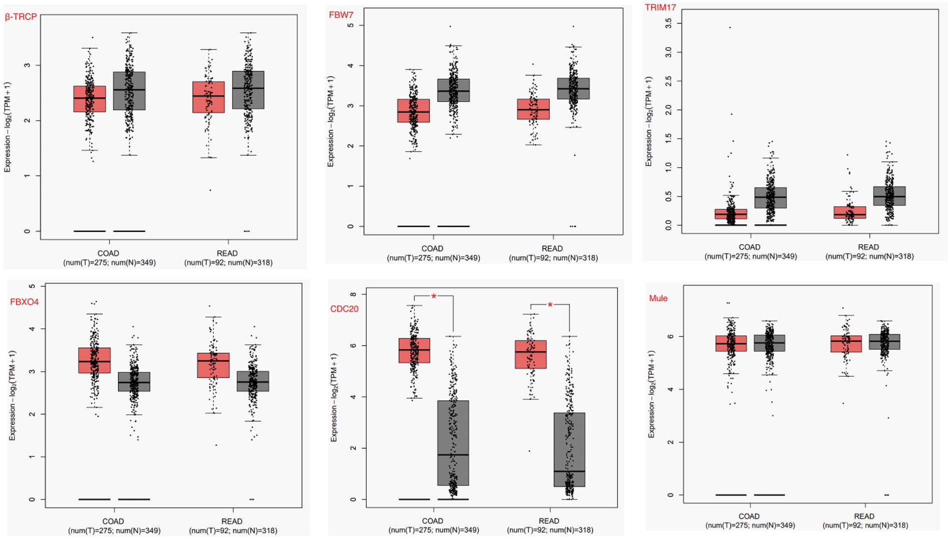

B

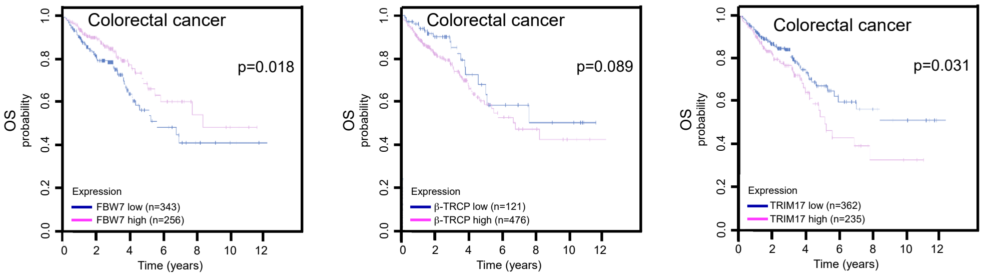

Supplementary Figure S3

A

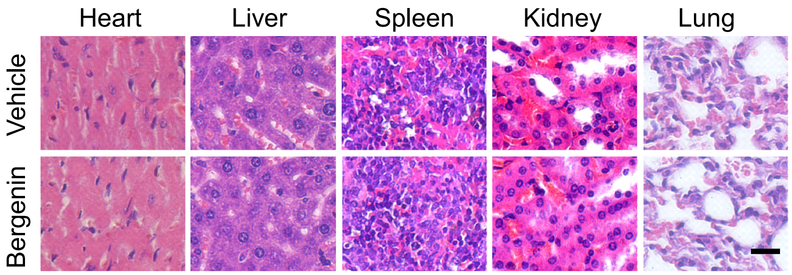

Supplementary Figure S4

4-1 full gel for Figure 2

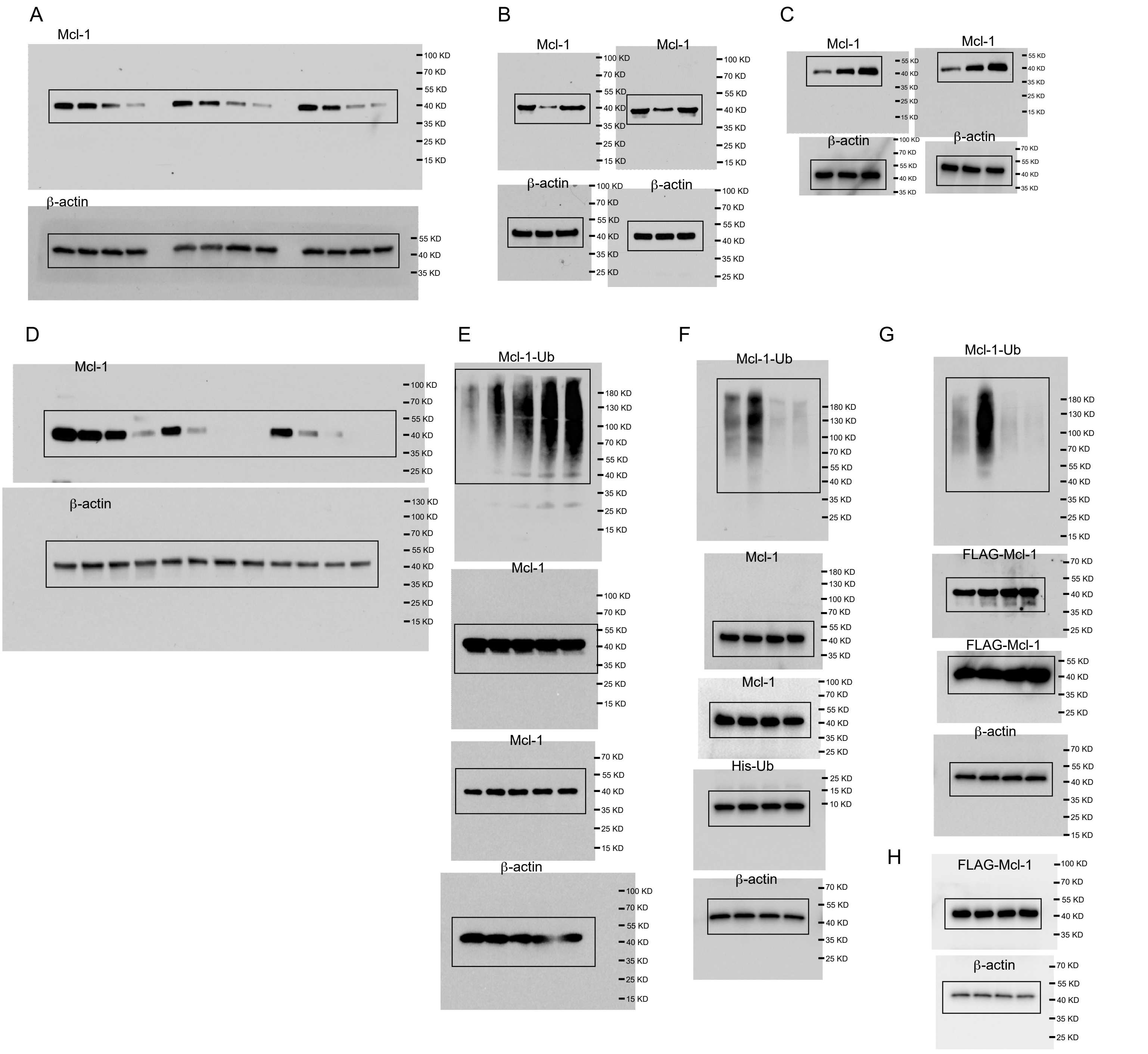

Supplementary Figure S4

4-2. full gel for Figure 3

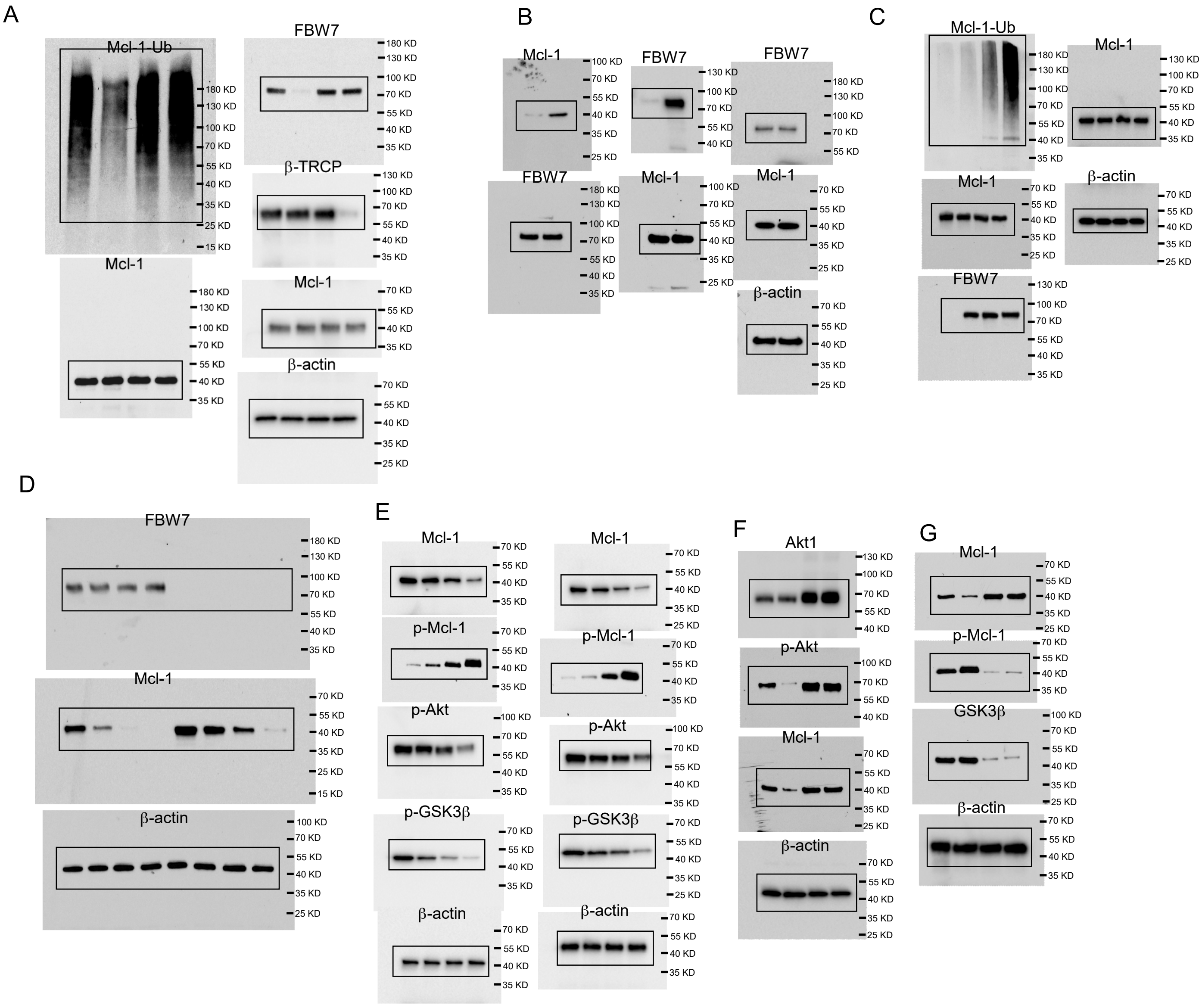

4-3. full gel for Figure 4

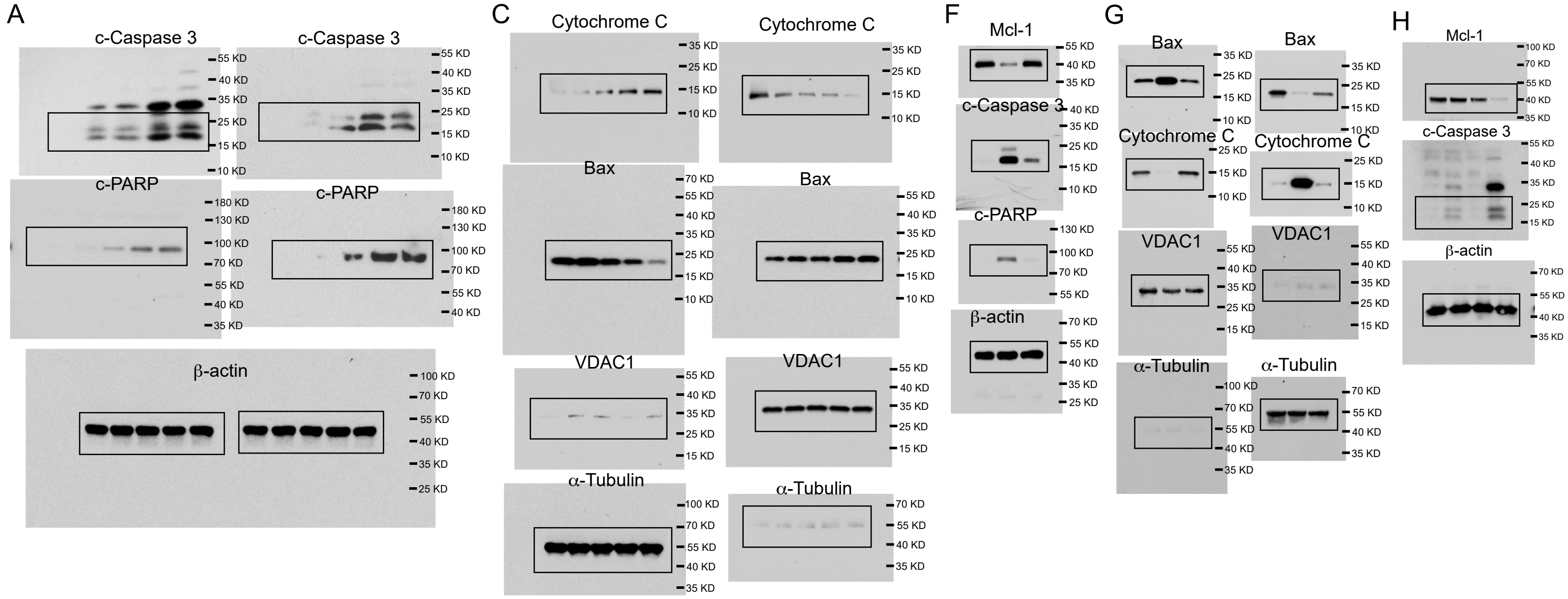

4-4. full gel for Figure 5B

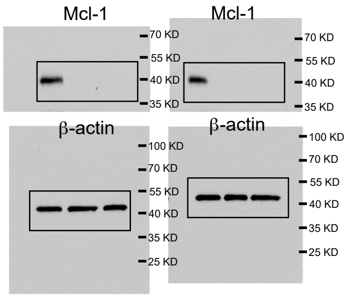

4-5. full gel for Supplementary Figure S1

B

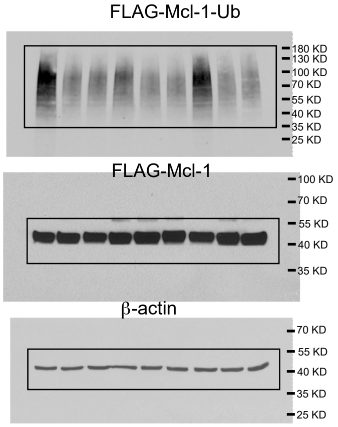

C

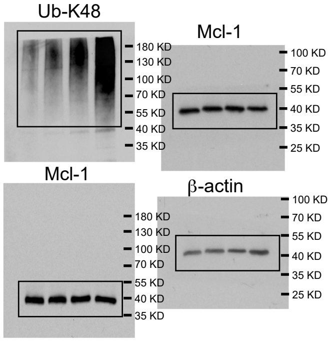

Supplement: Supplementary file 1 [file pharmaceuticals-16-00241-s001.zip › pharmaceuticals-2119632-supplementary.pdf]
